# Towards a better understanding of the nomenclature used in information-packaging efforts to support evidence-informed policymaking in low- and middle-income countries

**DOI:** 10.1186/1748-5908-9-67

**Published:** 2014-06-02

**Authors:** Taghreed Adam, Kaelan A Moat, Abdul Ghaffar, John N Lavis

**Affiliations:** 1 Alliance for Health Policy and Systems Research, World Health Organization, 1211 Geneva 27, Geneva, Switzerland; 2 Health Policy PhD Program and Program in Policy Decision-making, Centre for Health Economics and Policy Analysis, McMaster University, Hamilton, Canada; 3 McMaster Health Forum, Centre for Health Economics and Policy Analysis, Department of Clinical Epidemiology and Biostatistics, and Department of Political Science, McMaster University, Hamilton, Canada; 4 Department of Global Health and Population, Harvard School of Public Health, Boston, MA, USA

**Keywords:** Policy briefs, Health systems, Health policy, Synthesis, Summaries, Policymaking, Evidence-based

## Abstract

**Background:**

The growing recognition of the importance of concisely communicating research evidence and other policy-relevant information to policymakers has underpinned the development of several information-packaging efforts over the past decade. This has led to a wide variability in the types of documents produced, which is at best confusing and at worst discouraging for those they intend to reach. This paper has two main objectives: to develop a better understanding of the range of documents and document names used by the organizations preparing them; and to assess whether there are any consistencies in the characteristics of sampled documents across the names employed to label (in the title) or describe (in the document or website) them.

**Methods:**

We undertook a documentary analysis of web-published document series that are prepared by a variety of organizations with the primary intention of providing information to health systems policymakers and stakeholders, and addressing questions related to health policy and health systems with a focus on low- and middle-income countries. No time limit was set.

**Results:**

In total, 109 individual documents from 24 series produced by 16 different organizations were included. The name ‘policy brief/briefing’ was the most frequently used (39%) to label or describe a document, and was used in all eight broad content areas that we identified, even though they did not have obviously common traits among them. In terms of document characteristics, most documents (90%) used skimmable formats that are easy to read, with understandable, jargon-free, language (80%). Availability of information on the methods (47%) or the quality of the presented evidence (27%) was less common. One-third (32%) chose the topic based on an explicit process to assess the demand for information from policy makers and even fewer (19%) engaged with policymakers to discuss the content of these documents such as through merit review.

**Conclusions:**

This study highlights the need for organizations embarking on future information-packaging efforts to be more thoughtful when deciding how to name these documents and the need for greater transparency in describing their content, purpose and intended audience.

## Background

The availability of timely, suitably packaged and policy-relevant research evidence is important in supporting increased use of research evidence in the policy processes in low- and middle-income countries (LMICs) [1,2]. The growing recognition of the importance of developing concise materials to communicate various types of information to policymakers and those supporting them has underpinned the development of a plethora of information-packaging efforts, which aim to support action based on the messages arising from research and other policy-relevant information [1-3]. Furthermore, these packaging efforts are increasingly being buttressed by complementary ‘efforts to facilitate user-pull’—including clearinghouses, repositories or ‘one-stop shopping’—that seek to ensure quick and easy access to these documents by organizing them effectively (*e.g*., by type of content), making them more readily available in a single online space [3].

While the increased interest in the development of information-packaging efforts is undoubtedly welcome, it has led to wide variability in the types of products being prepared: those involved in their preparation are often based in different organizations, focused on different areas of health systems and policy research, working with different aims or intentions, and employing different approaches when packaging evidence. While variation and tailoring across contexts and intended audiences is to be expected—and, in fact, encouraged in many cases—the current state of the information-packaging field is at best confusing and fragmented, and at worst discouraging for those that they are intended to reach. In particular, the names used to describe the range of documents being prepared to inform policy are rarely used in consistent ways, and as such it is often difficult to determine what kind of information each document will contain, which can create added difficulty for those who are looking to benefit from them. This was recently highlighted in an attempt by a team of researchers to undertake a scoping review to highlight what is known from evaluations about three particular packaging mechanisms for systematic reviews: user-friendly summaries, policy briefs, and overviews [4]. The authors found that products utilizing these names all varied in their features and target audiences, and the same names were often used in the literature to refer to very different packaging mechanisms [4].

The impetus for this study grew out of the experience of one organization, the Alliance for Health Policy and Systems Research (AHPSR), trying to retrieve, archive, and make more accessible a range of documents aimed at supporting health systems policymakers and those supporting them through the provision of optimally packaged research evidence and related information. From an early stage in the process, it became clear to the team that grouping and presenting them in a way that promoted easier access was a difficult task. This was a result of the fact that, much as Chambers *et al*. found in their scoping review [4], there did not appear to be a common understanding and consistent nomenclature used across organizations and documents. Ironically, this lack of consistency and confusion may undermine attempts by policymakers, those supporting policymakers, and researchers to quickly access optimally packaged policy-relevant information when they need it most. Given that timeliness is consistently found to increase the likelihood that research evidence will be used in the policy process [5], the barrier that can result from this confusion should be seen as a significant challenge in efforts to support the use of research evidence and other policy-relevant information for health system strengthening.

As such, this study pursued two main objectives: to develop a better understanding of the range of names used by organizations preparing these documents to label them (*i.e*., title of series on the document itself) and describe them (*i.e*., in the text of the document or on the website); and to assess whether there are any consistencies in the characteristics and type of content of sampled documents across the names employed to label and describe them. We view this as a first step towards a more comprehensive understanding and consistent use of extant names that ultimately will help to reduce confusion and improve the prospects for timely access and retrieval of documents developed to inform policymaking processes in LMICs.

## Methods

We undertook an analysis of documents that are prepared with the primary intention of providing information (both research evidence and other types of policy-relevant information) to health systems policymakers, addressing questions related to health policy and health systems research. Our document sampling strategy was purposive and had two stages. First we used pilot work undertaken at the AHPSR to identify organizations that prepare series of policy-relevant documents (*e.g*., summaries and/or syntheses of research evidence and other policy-relevant information), that broadly aim to support health systems policymaking. This initial list of organizations was developed based on an electronic Google search that consisted of a combination of names that aimed to identify the types of products these organizations might produce to support policymakers (*e.g*., ‘brief’ , ‘briefing’ , ‘briefing notes’ , ‘evidence brief’ , ‘policy brief’ , ‘summary’ , ‘technical brief’ , ‘research summary’), as well as some of the core areas of health systems work that these organizations may be involved in (*e.g*., ‘human resources for health’ , ‘health financing’ , ‘governance’ , ‘health policy and systems research’ , ‘health policy’ , ‘health systems’).

In addition, we visited the booths of the organizations that exhibited their products at the First Global Symposium on Health Systems Research held in Montreux, Switzerland in November 2010, and collected samples of all relevant materials that fit the scope of this study, as described above. New names were identified during these first processes, and were used to broaden the initial Google search in an iterative manner until a diverse and sufficiently comprehensive list of organizations was documented. The resulting list was reviewed and supplemented by the knowledge gained through experience working in the field of health policy and systems research among staff working at the AHPSR and in identifying, packaging, and facilitating the retrieval of research evidence among staff at the McMaster Health Forum. We sought to achieve maximum variation with respect to the locations, organizational characteristics, and country focus of the organizations identified [6]. Our inclusion criteria for organizations were as follows:

1. Funds, conducts or disseminates research.

2. Focuses (at least in part) on health policy and systems research.

3. Focuses (at least in part) on studies and questions related to low- and middle-income countries.

4. Identifies (either implicitly or explicitly) policymakers as being among the target audience for their work.

5. Makes its publications freely available online.

6. Has prepared (or is currently preparing) a series, having prepared at least five documents or more that seek to inform and support the policymaking process (with no time limit).

Once organizations (and their document series) were identified, we sampled the five most recent documents prepared for each series, concluding our sampling at the end of 2012. When an institution had more than one series, we included samples from the other series if they appeared relevant. In the event that one of these series produced less than five documents, we included as many as were available. Although we initially intended to randomly sample five documents from each series, it was found that some series had changed significantly since inception, in terms of series content and style, which may render this sampling approach unfair for some of them given the novelty of their practice and the expected adjustments due to learning from earlier experiences. Our approach of using the most recent documents was adopted to ensure we had a consistent approach across all organizations’ document series. The search was conducted in English. No time limit was set.

In order to facilitate the analysis of the retrieved documents, as well as the development of an understanding of document characteristics, one investigator (KM) extracted data from each document using a template, which included the following information: year of publication, duration of series, number of pages (excluding appendices), number of documents produced since series inception, nomenclature used explicitly to label or describe the document, country (ies) that served as the focus of the document, whether the document described the methods it employed, whether the document explicitly drew on research evidence, and whether it provided recommendations for action. In addition, KM developed an annotated summary of each individual document, as well as for the document series as a whole to be used to analyse emergent themes across the retrieved documents. Next, a list of variables to describe and classify the documents’ content and characteristics (Table 1) was adapted from the criteria for communicating clearly developed by the BRIDGE study team [7] and informed by our understanding of some of the characteristics that have shown to be appreciated by end-users of summary documents (and other documents that aim to support health policy decision-making) in the literature [2,5]. Each series was reviewed according to this list of variables and given as ‘yes’ or ‘no’ code for each of them. TA independently reviewed all the retrieved documents and their coding. Minor discrepancies were noted and were resolved through discussion and consensus between the two analysts.

**Table 1:** Characteristics of retrieved documents

**Category**	**Criteria**	**Number N = 109**		**Percent = yes**
		**Yes**	**No**	
**What it covers**	**Topical/relevant issue** from the perspective of policy makers with an explicit process for determining topically/relevance (*e.g*., priority setting exercise, rapid response service).	35	74	32%
	**Document explicitly addresses at least four or more of the following:** political and/or health system contexts, problem, options, implementation considerations, and cost implications.	67	42	61%
**What it includes**	**Draws on synthesized/assessed research evidence** that has been assessed for its local applicability.	39	70	36%
	**Incorporates the tacit knowledge of policymaker/stakeholders** that has been collected in a systematic way and reported in a transparent manner.	20	89	18%
**For whom its targeted**	**Explicitly targets policymakers/stakeholders** as the key audience.	72	37	66%
	**Engages policymakers/stakeholders in merit review.**	21	88	19%
**How its packaged**	**Organized to highlight decision relevant information.**	72	37	66%
	**Understandable/lay language used.**	87	22	80%
	**In format that is readily appreciated** (*e.g*., graded entry).	98	11	90%
**How use is supported**	**Contextualized through online commentaries/briefings** provided by policymakers/stakeholders.	5	104	5%
**Features and content**	**Equity considerations** discussed or implicitly considered, *e.g*., through topic or analysis.	36	73	33%
	**Recommendations provided.**	47	62	43%
	**Methods described.**	51	58	47%
	**Quality of research evidence and/ or limitations outlined.**	29	80	27%
	**Reference list provided.**	84	25	77%
	**Local applicability discussed**, including case examples to highlight how a particular policy might be adapted to local circumstances.	44	65	40%
	**Key messages or summary points provided.**	63	46	58%

Adapted from [7] and informed by our understanding of useful characteristics appreciated by end users of summary documents [2,5].

Analysis of the sampled documents’ content began during initial data extraction, and consisted of an inductive thematic approach that proceeded in multiple iterations to classify the documents into different content types and develop a clear understanding of the purpose, audience, content and characteristics of these documents. Each cycle involved individual analysis by both investigators, followed by a face-to-face and phone conversations to discuss and refine emergent themes, and the relationship between these themes and the data being extracted from sampled documents. This process was also used to adapt and consolidate our understanding of the coding for each of the list of variables presented in Table 1, as described above. No follow-up was pursued with producers of documents to clarify aspects of our analysis, given that our intent was to assess each document in the same state that it had been made publicly available to intended users.

## Results

### Description of the retrieved documents

In total, 109 individual documents from 24 series produced by 16 different organizations were included in the analysis (Table 2). Out of the 24, six (35%) used ‘policy brief’ as the name of the series. Forty two percent of the documents focused on one or more specific countries, while 58% had an international focus (results not shown in tables). Some documents were prepared as outputs of multi-year grants that have since concluded (*e.g*., the Capacity Project), or as one-off series such as the policy briefs of the World Health Report 2006, while others seem to be part of an ongoing project of synthesis production such as the WHO Reproductive Health Library Commentaries, the SUPPORT Summaries and the Partnership for Maternal Newborn and Child Health (PMNCH) Knowledge Summaries. The length of documents ranged from 1 to 42 total pages (excluding appendices), with an average length of 9.5 pages and a median of 4 pages. All the sampled documents were available in English; 23 documents were also available in one or more other languages in addition to English, namely Arabic, French, German, Portuguese, Russian and Spanish. Most series were recent, having been initiated in 2005 or later, and only one was older, having been started in 2000. Series productivity, measured by the number of documents produced since the series’ inception, ranged from 1 to 132 and was not a reflection of the time since the series’ inception. The three most productive series started in 2005, 2007 and 2008 respectively.

**Table 2:** Document series included in the study

**Name of producing organization**	**Series name**	Number of documents sampled**	**Number of documents in the series since inception**	**Series duration**	**Average number of pages excluding appendices in the sampled documents (median)**
Capacity Plus	Issue Briefs	5	6	2011-present	2
Consortium for Research on Equitable Health Systems (CREHS)	Briefing Notes	1*	1	2007	1
Consortium for Research on Equitable Health Systems (CREHS)	Policy Briefs	5	24	2007-present	3.6
Consortium for Research on Equitable Health Systems (CREHS)	Briefing Sheets	1*	1	2010	4
EVIPNet/SURE	Evidence Briefs for Policy	5	11	2011-present	28.8
Global HIV/AIDS Initiatives Network (GHIN)	Policy Briefs	5	19	2009-present	8.4
Health Action International (HAI)	Briefing Papers	5	8	2000-2009	6.4
Health evidence Network/European Observatory on Health Systems and Policies (HEN-OBS)	Policy Briefs	5	15	2008-present	29.4
Human Sciences Research Council (HSRC)	Policy Briefs	5	16	2008-present	4.6
International Initiative for Impact Evaluation (3iE)	Briefs	5	19	2009-present	4
International Initiative for Impact Evaluation (3iE)	Evidence Matters	2*	2	2009-present	4.5
International Initiative for Impact Evaluation (3iE)	Systematic reviews	5	8	2009-present	2.4
IntraHealth International/Capacity Project	Legacy Series	5	11	2009	4
IntraHealth International/Capacity Project Knowledge Sharing	Technical Briefs	5	6	2006-2009	4.4
McMaster Health Forum	Evidence Briefs	5	11	2009-present	36
McMaster Health Forum	Issue Briefs	5	15	2009-present	28.6
Partnership for Maternal, Newborn and Child health (PMNCH), hosted by World Health Organization	Knowledge Summaries: Women's and Children's Health	5	22	2010-present	4
Social, Technological and Environmental Pathways to Sustainability Centre (STEPS)	Briefings	5	45	2007-present	2
Supporting Policy Relevant Reviews and Trials (SUPPORT)	SUPPORT Summaries	5	40	2008-present	7.2
World Bank: Reaching the Poor Series	Policy Briefs	5	6	2008	5.6
World Health Organization: Department of Health Systems Financing	Technical Briefs for Policymakers	5	15	2005-2010	8.2
World Health Organization: Department of Human Resources for Health	Spotlight	5	9	2006-2009	2
World Health Organization: Reproductive Health Library (RHL)	Commentaries	5	132	2005-present	4.4
World Health Organization: World Health Report 2006-Working Together for Health	Policy Briefs	5	6	2006	3.75
**ALL DOCUMENTS**		**109**	**448**		**9.5 (4)**

*When an organization had more than one series with one of them having five or more documents, we included samples from the other series even if they produced less than five documents for these series. **Sampling documents was completed in 2012.

Despite the fact that there were only 17 series names in the 24 series we retrieved (since, as Table 2 shows, six were called policy briefs), the results presented in Table 3 show that our sample revealed a total of 26 names that were used to either label or describe the documents included in our sample (within the document or the organization’s web page leading to the document series). Again, the most commonly used name was ‘policy brief/briefing’ , which was used in 39% of the sampled documents, followed by ‘brief/briefing’ (13%), and ‘issue brief’ (9%). In general, series that tended to use more than one name to label or describe their documents were also less consistent in the way the document was structured or the type of content the series covered and they did not usually provide a clear description of the purpose or nature of the content of the series; in comparison with those that consistently used a single name who generally paid much more attention to uniformity in style, content, formatting and intended use of these documents, notably the Joint HEN-OBS Policy Brief series, McMaster Health Forum Evidence Briefs and Issue Briefs, PMNCH Knowledge Summaries, and the SUPPORT Summaries.

**Table 3:** Names used in sampled documents

Names used to label or describe documents (not mutually exclusive)*	**Percentage (and number) of sampled documents that use the name to label or index the document (not mutually exclusive)**	**Number of different series that use the name to label or index documents**
Policy brief/briefing	39% (42)	11
Brief/briefing	13% (14)	4
Issue brief	9% (10)	2
Briefing paper	6% (7)	3
Evidence brief	6% (6)	1
Commentary	5% (5)	1
Legacy series	5% (5)	1
Knowledge Summary	5% (5)	1
Spotlight	5% (5)	1
SUPPORT summary	5% (5)	1
Systematic review	5% (5)	1
Technical brief	5% (5)	1
Technical brief for policy-makers	5% (5)	1
Evidence-based policy brief	4% (4)	1
Response	4% (4)	1
Evidence matters	2% (2)	1
Research brief	2% (2)	1
Briefing note	1% (1)	1
Briefing sheet	1% (1)	1
Domain briefing	1% (1)	1
Enduring questions brief	1% (1)	1
Evidence brief for policy	1% (1)	1
Evidence in Brief	1% (1)	1
Impact evaluation	1% (1)	1
Project briefing	1% (1)	1
Study findings and recommendations	1% (1)	1

### Content types

Our analysis also resulted in the identification of eight broad content types that were found to characterize the documents in our sample (Table 4). We also mapped the range of names used to label or describe these documents by content type as well as the frequency by which each content type was represented in our sample. Overall, we found little to no consistency in the names used to describe documents with similar content, with some documents with the same content type being labelled or described with up to 10 different names. Many names—and in particular ‘policy brief’—were used across many content types, while some (*e.g*., ‘project briefing’) were only used in one document series. In fact, ‘policy brief’ was used in each of the eight different content types that we identified. This analysis suggested that there were no obviously common traits among documents that were referred to using the same name, and it wasn’t clear why some organizations used one name to refer to their documents (*e.g*., policy brief) instead of another name (*e.g*., technical brief).

**Table 4:** How names were used to label or describe various types of documents in the sample

**Content of sampled documents**	**Number of sampled documents with this content area (%)**	**Number of names used to label or describe documents within content areas**	Names used to label or describe document types (times used)*
1. Essays, background papers or commentary about a specific topic (draws on evidence but not prepared as a literature review on the topic)	29 (27%)	10	Policy brief/briefing (8)
			Issue brief (5)
			Knowledge summary (5)
			Spotlight (5)
			Brief/Briefing (3)
			Evidence matters (2)
			Technical brief for policy-makers (2)
			Impact evaluation (1)
			Technical brief (1)
			Project briefing (1)
2. Synthesis that starts with a priority policy issue in a specific jurisdiction, and then draws on a range of research evidence (prioritizing systematic reviews) to inform the problem underlying the issue, options for addressing it and implementation considerations (*e.g*., overview of reviews)	20 (18%)	5	Policy brief (10)
			Evidence brief (6)
			Issue brief (5)
			Evidence-based policy brief (4)
			Evidence brief for policy (1)
3. Formal literature review of what is known about a particular policy domain (draws on different types of evidence that was systematically identified, selected and appraised)	18 (17%)	7	Policy brief (15)
			Briefing paper (1)
			Briefing/brief (3)
			Domain briefing (1)
			Enduring questions brief (1)
			Study findings and recommendations (1)
			Technical brief (1)
4. Summary of a research synthesis that answers a single question (*i.e*., summary of a systematic review)	18 (17%)	7	Commentary (5)
			SUPPORT summary/Summary (5)
			Systematic review (5)
			Evidence matters (2)
			Policy brief (2)
			Briefing (1)
			Briefing note (1)
5. Summary or lessons learned from a particular program or policy in a specific context (including the results of M&E)	14 (13%)	9	Policy brief (5)
			Legacy series (5)
			Briefing/brief (4)
			Technical brief for policy-makers (3)
			Research brief (2)
			Briefing sheet (1)
			Briefing paper (1)
			Evidence in Brief (1)
			Impact evaluation (1)
6. Opinion pieces and organizational position statements (does not draw on research evidence systematically)	11 (10%)	7	Briefing paper (5)
			Legacy series (5)
			Brief (4)
			Response (4)
			Policy brief (1)
			Evidence in brief (1)
			Technical brief (1)
7. Integration of lessons learned from particular programs or policies in specific contexts with relevant literature	3 (3%)	2	Technical brief (2) Policy brief (1)
8. Summary of a single study (or related single studies)	1 (1%)	1	Policy brief (1)

*It is important to note that one document could have used more than one name so that the total number of names used to label or describe documents in not mutually exclusive and may be higher than the total number of sampled documents in each content area (column 2). For example, in the first row, while 29 of the sampled documents corresponded to this type of content, 33 names were used to describe or label them.

### Document characteristics

Table 1 shows the main features and characteristics of the sampled documents. Most documents (90%) used a skimmable format that is easy to read; (*e.g*., using a graded entry format) [2]; used understandable jargon-free language (80%); and were organized in such a way to highlight decision relevant information (66%). In terms of content, a high proportion included a reference list (77%) and provided a list of key messages (58%). However, fewer described the methods (47%) and the quality of the evidence presented (27%). Around 36% drew on synthesized evidence from systematic reviews.

While many documents (61%) covered at least four aspects of the topic addressed (*e.g*., political and/or health system contexts, problem, options, implementation considerations, cost implications), fewer documents (32%) assessed policymakers’ demand for information on the selected topic, or engaged with them to discuss the content of the document, *e.g*., as part of a merit review process (19%). The majority relied on web-based dissemination and only few used online contextualization or commentaries provided by end-users (5%).

## Discussion

Our analysis of 109 documents prepared within 24 different series suggests that there is currently little consistency in the names used to label or describe types of documents that have similar contents, and the use of a discrete set of names to help differentiate between documents that have different contents is similarly rare. We also found that there was inconsistent use of names used to refer to documents that are part of the same series and characterized by similar content—while the 24 series only used 17 names to label the series on the document itself, 26 names were used to describe or label them either on the organization’s web-page that provides links to these documents or within the documents themselves. In particular, ‘policy brief’ was used very loosely when referring to documents with a different label on the document itself.

However, we did observe some alignment in the names, content, and style used in some series, particularly those prepared to support the use of systematic reviews in policymaking —*e.g*., the SUPPORT Summaries and the McMaster Health Forum Evidence Briefs series; and those presenting commentaries or essays on a particular topics, *e.g*., the PMNCH Knowledge Summaries and the Joint HEN-OBS Policy Brief series. Furthermore, these series made explicit the purpose and content of their documents, and in some cases also linked the names used to label or describe them with their characteristics. These series were also consistently uniform in their content and formatting, which served to establish a more solid understanding of what they include, aside from making the whole series much easier and faster to read.

While the EVIPNet/SURE Project Policy Briefs also provide readers with information on the type of content and intended use (which is undoubtedly helpful to make clear what their documents provide readers), and are also consistent in their content and formatting, they were less consistent in their use of names to label or describe the document. This is likely the result of the fact that the series has evolved over time based on group feedback, consists of documents prepared by multiple organizations located in a variety of countries, and are prepared by organizations operating in a number of languages. As we have suggested earlier, ignoring these types of discrepancies (which were found in many of the document series’ sampled), can only serve to muddy the waters for those who need the policy-relevant information contained in this type of synthesis in a timely way. It also makes it difficult to retrieve the relevant information (due to multiple nomenclatures), thereby reducing the likelihood that they will serve to perform their main function.

While many sampled documents were found to have several helpful features (*e.g*., content that is easy-to-read, jargon free, and referring the reader to more reading and reference material), there is room for improvement. Specifically, more documents could consider describing the methods used to prepare them, reporting the quality of research evidence included or summarized in them, engaging with policy makers at different stages of their development (including in assessing the demand for new evidence), and using more proactive ways to disseminate them [7,8].

There are three main strengths of this study. First, this is to our knowledge the first explicit attempt to map the nomenclature used by various organizations to label or describe the documents prepared to support the policymaking process. While there has been some recent work undertaken in Europe that aims to take stock of the various approaches to packaging information [7], we explicitly sought to make sense of the language being used to label or describe these documents as a way to promote greater clarity among potential users. Second, our sampling of documents was largely underpinned by knowledge of those who have worked in the field (at the Alliance). As such, our approach ensured we included documents from series that are widely known by policymakers and those supporting them, but are not likely indexed in major electronic databases. Relying on electronic searches may have led to us overlooking some important documents in our analysis. Given our results, it is also likely that the observed inconsistencies in nomenclature would have made it very difficult to find documents using traditional search strategies based on key names. Finally, that we were able to analyze 109 documents from 24 different series suggests that, although not comprehensive, our analysis has provided rich insights and a compelling account of the use of nomenclature to label and describe documents that are prepared to inform health system policymaking.

Our study also has three limitations. First, given resource constraints we could not hope to be completely comprehensive with respect to the documents sampled for analysis. It is a distinct possibility that we have left out a document series that could have provided useful insights about the best way to use the names found in this study. Second, despite their important role assisting the analysis, the categories that emerged and were used to classify documents in terms of content type are not as individually distinct as we would have liked. This suggests that a different pair of analysts may have a different interpretation of a particular document’s characteristics—particularly when the authors or organizations presenting them don’t provide any details about their content (and this was often the case). Third, we used an adapted version of the BRIDGE criteria to assess a range of different document types, and it is possible that the metrics used aren’t as applicable to some producers and document types (or content types) as they are to others. However, given our interest in this paper was to assess documents that have the goal of providing information as an input into the policymaking process, and the characteristics captured have been found to be the most promising characteristics of information-packaging mechanisms that aim to achieve this purpose, we felt it appropriate to use these criteria across the documents sampled. Finally, while not necessarily a weakness, it should also be highlighted that our analysis drew primarily on documents focused on health policy and systems (or fields directly related as was the case with the STEPS documents). As such, there may be other disciplines or sectors that could contribute to our understanding, but have been missed here.

## Conclusion

While this study has painted a picture of confusion and complexity rather than providing clarity, two main lessons can be gleaned from our results. First, despite its inherent attractiveness among those seeking to engage with the policy process, the name ‘policy brief’ has become so ubiquitous that it may no longer serve as a useful signal to potential users about what can be expected in a document: this study found it being applied to eight very different content types. As such, it may serve to distract a user from seeking out helpful information, particularly if they retrieve many irrelevant documents labelled ‘policy brief’ before finding exactly what they are looking for. It also complicates the task among those attempting to create ‘one-stop shopping’ for these documents, particularly because the name does not provide any useful indications about how to organize them online for easy, timely access. If the name must be used, we encourage those employing it to provide explicit details about document aims and content.

Secondly, and perhaps most importantly, it seems extremely useful that organizations preparing documents that have as their intention to support policymaking processes strive to be more descriptive when deciding which names they will use to label or describe these documents. Making it clear to their audience what the document intends to cover and who are the target audiences will also help to promote greater clarity with respect to the purpose, contents and formats that can be expected within each document type (Table 1). Series such as the ‘SUPPORT summaries’ used by the SUPPORT group or the ‘evidence briefs’ used by the McMaster Health Forum provide an illustration of how useful consistency is.

Overall, the steep growth in the development of more useful, relevant, timely, and optimally packaged documents that aim to support the use of research and other policy-relevant information in the policy process, is certainly positive. The findings from this study illustrate the potential usefulness of a move towards greater consistency in the names used to label the different types of documents and greater clarity about what their purpose, content and intended audience.

## Competing interest

The AHSPR funded some of the projects that resulted in the preparation of documents included in this analysis, namely SUPPORT summaries and EVIPNet series, and two of the authors (KAM and JNL) are involved with EVIPNet and McMaster Health Forum projects (including the preparation and evaluation of evidence briefs). JNL is also co-editor of the HEN/OBS series.

## Authors’ contributions

TA, KAM and JNL conceptualized the paper and developed the methods. KAM and TA performed the analysis and interpretation of the results and drafted the paper. JNL and AG contributed to the interpretation of the results and identification of relevant organizations and series. All authors read and approved the final manuscript.

## References

[B1] Lavis JN How Can We Support the Use of Systematic Reviews in Policymaking? PloS Med 2009 6 11 e1000141 doi:10.1371/journal.pmed.1000141 10.1371/journal.pmed.1000141 19936223 PMC2777391

[B2] Rosenbaum SE Glenton C Wiysonge CS Abalos E Mignini L Young T Althabe F Ciapponi A Marti SG Meng Q Wang J la Hoz Bradford AM Kiwanuka SN Rutebemberwa E Pariyo GW Flottorp S Oxman AD Evidence summaries tailored to health policy-makers in low- and middle-income countries Bull World Health Organ 2011 89 1 54 61 10.2471/BLT.10.075481 21346891 PMC3040014

[B3] Lavis JN Lomas J Harnid M Sewankambo NK Assessing country-level efforts to link research to action Bull World Health Organ 2006 84 620 628 10.2471/BLT.06.030312 16917649 PMC2627430

[B4] Chambers D Wilson PM Thompson CA Hanbury A Farley K Light K Maximizing the Impact of Systematic Reviews in Health Care Decision Making: A Systematic Scoping Review of Knowledge-Translation Resources Milbank Q 2011 89 131 156 10.1111/j.1468-0009.2011.00622.x 21418315 PMC3160597

[B5] Lavis J Davies H Oxman A Denis JL Golden-Biddle K Ferlie E Towards systematic reviews that inform health care management and policy-making J Health Serv Res Policy 2005 10 Suppl 1 35 48 10.1258/1355819054308549 16053582

[B6] Patton MQ Qualitative Research and Evaluation Methods 2002 Thousand Oaks, Canada: Sage

[B7] Lavis JN Catallo C Permanand G Zierler A BRIDGE Study Team BRIDGE Summary 1: Communicating Clearly: Enhancing Information-Packaging Mechanisms to Support Knowledge Brokering in European Health Systems 2013 Brussels, Belgium: European Observatory on Health Systems and Policies Ref Type: Report

[B8] Lavis JN Permanand G Oxman AD Lewin S Fretheim A SUPPORT Tools for evidence-informed health Policymaking (STP) 13: Preparing and using policy briefs to support evidence-informed policymaking Health Res Policy Syst 2009 7 S13 10.1186/1478-4505-7-S1-S13 20018103 PMC3271824

